# Delivery Room Intensive Care Unit: 5 Years' Experience in Assistance of High-Risk Newborns at a Referral Center

**DOI:** 10.3389/fped.2021.647690

**Published:** 2021-04-29

**Authors:** Silvia Buratti, Elisabetta Lampugnani, Monica Faggiolo, Isabella Buffoni, Dario Paladini, Gabriele De Tonetti, Giulia Tuo, Maurizio Marasini, Girolamo Mattioli, Andrea Moscatelli

**Affiliations:** ^1^Neonatal and Pediatric Intensive Care Unit, Department of Critical Care and Perinatal Medicine, Istituto di Ricovero e Cura a Carattere Scientifico (IRCCS) Istituto Giannina Gaslini, Genova, Italy; ^2^Fetal Medicine and Surgery Unit, Department of Critical Care and Perinatal Medicine, Istituto di Ricovero e Cura a Carattere Scientifico (IRCCS) Istituto Giannina Gaslini, Genova, Italy; ^3^Obstetric Anesthesia, Department of Critical Care and Perinatal Medicine, Istituto di Ricovero e Cura a Carattere Scientifico (IRCCS) Istituto Giannina Gaslini, Genova, Italy; ^4^Pediatric Cardiology and Cardiac Surgery, Department of Surgery, Istituto di Ricovero e Cura a Carattere Scientifico (IRCCS) Istituto Giannina Gaslini, Genova, Italy; ^5^Dipartimento di Neuroscienze, Riabilitazione, Oftalmologia, Genetica e Scienze Materno-Infantili, University of Genova, Genova, Italy; ^6^Paediatric Surgery Unit, Department of Surgery, Istituto di Ricovero e Cura a Carattere Scientifico (IRCCS) Istituto Giannina Gaslini, Genova, Italy

**Keywords:** high-risk newborn, congenital heart disease, congenital diaphragmatic hernia, delivery room, intensive care

## Abstract

**Objective:** The aim of the study is to describe a delivery room intensive care unit (DRICU) model and evaluate its effectiveness in preventing morbidity and mortality in high-risk newborns.

**Design:** This retrospective case series includes all DRICU procedures performed from 2016 to 2020.

**Setting:** Gaslini Children's Hospital is a major pediatric tertiary care center where high-risk pregnancies are centralized. The Neonatal and Pediatric Intensive Care Unit admits every year about 100 high-risk newborns.

**Patients:** The selected patients are newborns at risk of critical conditions immediately after birth for respiratory or cardiovascular congenital disorders.

**Interventions:** The perinatal plan is defined by the multidisciplinary team of Fetal and Perinatal Medicine. The DRICU procedure provides highly specialized care through a protocol that includes logistics, personnel, equipment, and clinical pathways.

**Main Outcome Measures:** The primary outcome is the prevention of acute complications and mortality in the delivery room and early neonatal period.

**Results:** From 2016 to 2020, 40 DRICU procedures were performed. The main prenatal diagnoses included congenital heart disease with a high risk of life-threatening events immediately after birth (38%), congenital diaphragmatic hernia (35%), and fetal hydrops/hydrothorax (23%). Mean gestational age was 35.9 weeks (range: 31–39), and mean birth weight was 2,740 grams (range: 1,480–3,920). DRICU assistance completed in all patients by neonatal intensivists included tracheal intubation and arterial and central venous cannulation; complex procedures such as ex-utero intrapartum technique and extracorporeal membrane oxygenation cannulation are described. No deaths nor severe acute complications occurred in the delivery room or in the immediate postnatal period.

**Conclusions:** The outcome in critical newborns is potentially affected by planned assistance strategies and specialized competencies through the implementation of a DRICU protocol.

## Introduction

The delivery room intensive care unit (DRICU) concept has been introduced by Vento et al. in 2008 as a practice to enhance the survival rates and reduce the morbidity of extremely preterm infants (1). The intention of DRICU is to initiate intensive care environment and technology in the delivery room (DR) to adequately provide care immediately after birth in critical newborns (1–3). The recommendations by Vento et al. included additional monitoring with pulse oximeter or electrocardiography, FiO_2_ titration, temperature control, and ventilation strategies to prevent lung injury in preterm infants (1, 2). As in extremely preterm newborns, the survival and morbidity rates in conditions like critical congenital heart disease (CHD) or congenital diaphragmatic hernia (CDH) are potentially affected by the clinical management in the first few minutes of life.

Over the past decade, our institution has revised and implemented the DRICU concept to include resuscitation protocols that require advanced equipment and highly specialized personnel for the assistance of critical newborns with complex conditions. Our aim is to describe the Gaslini Children's Hospital DRICU model since its full implementation in 2016 and evaluate its effectiveness in preventing morbidity and mortality in high-risk newborns.

## Methods

Gaslini Children's Hospital is a major pediatric public tertiary center that provides all pediatric subspecialty services to national and international referrals. The Neonatal and Pediatric Intensive Care Unit (ICU) admits every year about 100 high-risk newborns with complex congenital anomalies requiring general surgery, neurosurgery, cardiovascular surgery, or extracorporeal support. Only a limited number of these newborns are at risk of critical conditions immediately after birth and are selected for the DRICU procedure. In our center, the birth event is carefully planned through centralization of high-risk pregnancies and periodic case discussion in the multidisciplinary team of Fetal and Perinatal Medicine. The team includes several specialists: fetal medicine/surgery, obstetrics/gynecology, neonatal and pediatric ICU, neonatology, cardiology, cardiac surgery, general surgery, neurosurgery, nephrology, fetal and perinatal pathology, obstetric anesthesiology, genetics, radiology, neuroradiology, and psychology. The DRICU protocol includes details in planning, logistics, personnel, equipment (Table 1), and clinical pathways.

**Table 1:** Delivery room intensive care unit equipment.

**Equipment**	
Temperature control	Temperature Servo control isolette
Advanced monitoring	Pulse oximeter, end tidal CO_2_, EKG, invasive arterial pressure, central temperature, near-infrared spectroscopy
	Arterial blood gas analysis
Airways	Routine and difficult airway equipment
Ventilation	Neonatal ventilator
	High-frequency oscillatory ventilator
Circulation	Vascular access equipment for arterial and central venous catheterization
For specific procedures when indicated	Thoracic and peritoneal drains
	Equipment for balloon atrial septostomy
	Rigid bronchoscope for tracheal plug removal
Diagnostics	Portable ultrasound (US) for echocardiography, lung and abdominal US, and vascular access
	Portable X-ray
Medications	Fentanyl, midazolam, and rocuronium
	Epinephrine
	Prostaglandin E1
	Surfactant
	Inhaled nitric oxide

### Patient Selection

The patients include newborns at risk of critical conditions immediately after birth for respiratory or cardiovascular congenital disorders.

### Planning

Once all prenatal information has been gathered, a detailed resuscitation plan is defined. A Cesarean section is scheduled in the morning session during a weekday. DRICU can also be implemented, as an emergency, any time (24/7) if labor occurs in a known high-risk pregnancy (at term or preterm).

### Logistics

While regular deliveries occur in the Ob/Gyn Department, DRICU is set up in the Operating Room (OR) adjacent to the ICU. If required, the catheterization laboratory and/or one OR is kept on stand-by for emergent procedures.

### Intensive Care Personnel

Two intensive care physicians are assigned—one senior (team leader) and one junior assistant—or, alternatively, two or three senior physicians if complex procedures are planned, such as in ex-utero intrapartum treatment (EXIT) and extracorporeal membrane oxygenation (ECMO).

Two to three skilled neonatal nurses are selected.

The roles in resuscitation and procedures are precisely defined prior to delivery. Each team (one physician assisted by one nurse) is in charge of airways management or vascular access in the standard procedure.

### Clinical Pathways

Detailed prenatal assessment is crucial for case selection of high-risk newborns. Defined clinical pathways are followed depending on specific diagnoses. The standard procedure includes early elective intubation and arterial (umbilical, radial, or femoral) and central venous (umbilical or femoral) catheterization. Advanced monitoring is immediately started on the temperature Servo control isolette.

#### Congenital Heart Disease

Risk stratification systems of prenatally diagnosed CHD with recommended care plans have been published (4). In D-transposition of the great arteries (dTGA), DRICU is planned when fetal predictors of inadequate circulatory mixing are identified at last fetal echocardiogram (5). After DRICU assistance, the cardiologist may perform emergency echo-guided urgent balloon atrial septostomy (BAS) in the DR, or, more frequently, patients are immediately transferred to the cath lab for BAS. In other critical CHD diagnoses, after stabilization, the patients are immediately transferred to the OR for emergency surgical repair [e.g., obstructed total anomalous pulmonary venous return (TAPVR)] or to the cath lab for atrial septum perforation or BAS [e.g., hypoplastic left heart syndrome (HLHS) and restrictive atrial septum] or for other catheter-based emergency interventions. Cardiology and cardiothoracic surgery are notified prior to delivery.

#### Congenital Diaphragmatic Hernia

Ultrasound and MRI prenatal evaluations are used to define prognostic factors (hernia laterality, fetal observed-to-expected lung-area-to-head circumference ratio, total fetal lung volume, liver herniation, and stomach position) and to identify indications to fetoscopic endoluminal tracheal occlusion (FETO). At birth, the newborn is immediately intubated and connected to the ventilator, thus avoiding mask and manual ventilation. Sedation is obtained to optimize a “gentle” ventilation strategy with high-frequency oscillatory ventilation (HFOV). A gastric tube is inserted to decompress the stomach. Inhaled nitric oxide (iNO) and inotropic support are started, if indicated. The patient is transferred to the ICU. When tracheal occlusion is in place at time of delivery, the EXIT procedure is performed before the assistance previously described.

#### Hydrothorax/Fetal Hydrops

Severity of pleural, pericardial, and peritoneal effusions is evaluated with prenatal ultrasound.

The patient is immediately intubated and connected to HFOV while sedation is given and pleural, pericardial, and/or peritoneal drains are placed with ultrasound guidance. Inhaled NO and inotropic support are started, if indicated. The patient is transferred to the ICU.

#### Exit

EXIT involves the planned partial delivery of the fetus via hysterotomy while maintaining uterine relaxation and placental support, allowing for the establishment of neonatal cardiopulmonary stability in a controlled manner. The EXIT procedure can be implemented as EXIT-to-airway, EXIT-to-resection, or EXIT-to-ECMO. Interventions performed during EXIT may include endotracheal intubation, tracheostomy, mass excision, removal of a temporary tracheal occlusive device, and ECMO cannulation. The procedural details are described elsewhere (6).

In more complex cases, an individual plan is defined antenatally. All procedures aimed to stabilize the newborn are completed by the neonatal intensivists. Fetal procedures (i.e., pre-partum thoracentesis) are performed by the fetal medicine specialist. Other specialists (more frequently the cardiologist or the general surgeon) are present at delivery, as indicated by the diagnosis. After stabilization in the DRICU, the patient may be transferred to the OR for a surgical procedure, to the cath lab, or, more frequently, to the neonatal and pediatric ICU.

We performed a retrospective review of all DRICU cases performed at Gaslini Children's Hospital between 2016 and 2020. Data collection included prenatal diagnostic data, case discussion reports, perinatal procedures, clinical history, and outcome. Study approval was obtained from our Institutional Review Board. We used the CARE checklist when writing this report (7).

## Results

Patient diagnosis, weight, gestational age (GA), DRICU procedures and support, procedures and clinical course after DRICU assistance are reported in Table 2.

**Table 2:** Reported delivery room intensive care unit (DRICU) cases.

***N***	**Diagnosis**	**Weight (g)**	**GA (weeks)**	**Fetal anesthesia for prenatal procedure**	**DRICU procedures^a^ and support**	**Procedures after DRICU and outcomes**
		**Min–max** **(mean)**				
10	Left CDH Twin (1)	1,600–3,920 (2,780)	34–38	No	Standard (10) HFOV (10) iNO (5)	VV ECMO (2 + 1 with two runs) CRRT (1) Surgical hernia repair (10) DA closure (1) Tracheostomy (1) Exitus (1)
4	CDH with tracheal plug Left (2) Right (2)	1,890–2,800 (2,320)	31–36	Yes for EXIT and tracheal plug removal (2)	Standard (4) HFOV (4) iNO (3) epinephrine (2)	VV ECMO (3) CRRT (2) Surgical hernia repair (4) DA closure (2) Tracheostomy (1) Exitus (1)
6	Hydrothorax Trisomy 21 (1)	1,500–3,850 (2,350)	31–38	Yes for thoracentesis (4)	Standard (6) HFOV (5) Pleural drain (2) Epinephrine (1)	Bilateral pleural drain (3) Surgical exeresis of pulmonary sequestration (1) VSD-DA closure (1)
3	Hydrops Twin and monosomy X (1)	1,480–3,000 (2,400)	33–36	No	Standard (3) HFOV (3) Pleural drain (2) Peritoneal drain (1)	Bilateral pleural drain (2) CRRT (1)
8	dTGA restrictive FO	2,690–3,500 (3,100)	36–38	No	Standard (8)	BAS—arterial switch (8)
1	Obstructed TAPVR	2,900	38	No	Standard HFOV	Surgical correction
1	TAPVR, hydrothorax	2,300	39	No	Standard pleural drain epinephrine	Exitus during surgical correction (severe pulmonary veins hypoplasia)
1	HLHS restrictive atrial septum	3,300	38	No	Standard	RF atrial septum perforation and stent—hybrid procedure
1	HLHS restrictive atrial septum	3,450	39	No	Standard	BAS—I stage palliation
1	PAIVS and hydrothorax	2,220	34	Yes for thoracentesis	Standard HFOV	Atrial septostomy Stent DA CRRT Exitus
1	PAIVS and prenatal closure of DA	3,000	37	YES for Intrauterine PGE1 infusion	Standard VV ECMO epinephrine milrinone PGE1	BT shunt, failure Stent DA
1	Critical aortic valve stenosis restrictive FO	2,850	38	No	Standard	Percutaneous balloon valvuloplasty
1	Pulmonary hypoplasia due to bilateral renal dysplasia and anhydramnios	3,750	38	No	Standard HFOV	Bilateral nephrectomy VV ECMO CRRT Exitus
1	Intracerebral germ cell tumor and severe hydrocephalus	2,700	33	YES for cephalocentesis	Standard	Palliative care Exitus

a *Standard procedure: naso-tracheal intubation, arterial (umbilical, radial or femoral), and central venous (umbilical or femoral) catheterization*.

*BAL, balloon atrial septostomy; BT, Blalock Taussig; CDH, congenital diaphragmatic hernia; CRRT, continuous renal replacement therapy; DA, ductus arteriosus; EXIT, ex utero intrapartum technique; FO, foramen ovale; GA, gestational age; HFOV, high-frequency oscillatory ventilation; HLHS, hypoplastic left heart syndrome; iNO, inhaled nitric oxide; PAIVS, pulmonary atresia with intact ventricular septum; RF, radiofrequency; TAPVR, total anomalous pulmonary venous return; dTGA, D-transposition of great arteries; VV ECMO, veno-venous extracorporeal membrane oxygenation*.

### Patients

From January 2016 to June 2020, 40 DRICU procedures were performed at Gaslini Children's Hospital. The diagnoses included 35% CDH (14/40, four with previous tracheal plug placement), 23% fetal hydrops/hydrothorax (9/40, one with pulmonary sequestration), 20% isolated dTGA with restrictive foramen ovale (FO; 8/40), 5% severe HLHS with restrictive atrial septum (2/40), 5% pulmonary atresia with intact ventricular septum [PAIVS; 2/40, one with prenatal closure of ductus arteriosus (DA) and one with hydrothorax], 5% TAPVR (2/40, one obstructed and one with hydrothorax), one critical aortic valve stenosis with restrictive FO, one severe pulmonary hypoplasia in bilateral renal dysplasia, and one intracranial germ cell tumor with severe hydrocephalus. The mean GA was 35.9 weeks (range: 31–39), and the mean birth weight was 2,740 g (range: 1,480–3,920).

### Procedures

DRICU procedures included tracheal intubation and arterial and central venous cannulation in all patients (standard DRICU), HFOV in 25 patients (eight with iNO administration), and six thoracic/peritoneal drain placements. In six cases, when an *in utero* procedure was performed immediately before C-section, fetal anesthesia with neuromuscular blockade required full ventilatory support after birth. In two cases, EXIT was performed, respectively, at 31 and 32 weeks of GA for premature rupture of membranes in CDH fetuses with tracheal plug; the tracheal plug was removed by rigid tracheoscopy; the newborns were assisted as a DRICU procedure and then transferred to the ICU in stable condition. In one case of PAIVS and prenatal closure of DA, emergency veno-venous ECMO support with echoguided percutaneous bicaval double-lumen cannulation was established in the DR in 20 min. The technique was recently described by our group (8). This approach was defined because parents did not consent for EXIT. All patients with dTGA required an emergency BAS in the cath lab, as predicted prenatally. The two patients with HLHS and restrictive atrial septum were immediately transferred to the cath lab, one for radiofrequency perforation of the atrial septum and stent implantation and the other for BAS. One patient with critical aortic valve stenosis and restrictive FO underwent percutaneous balloon valvuloplasty in the cath lab. The patient with obstructed infracardiac TAPVR was immediately transferred to the OR for surgery.

### Outcomes

No deaths nor severe acute complications occurred in our patients in the DR. Only one case of bilateral pneumothorax occurred during DRICU management in a patient with fetal hydrops, which was promptly treated. No cases of pneumothorax occurred in patients with CDH or hydrothorax. No acute neurological complications were observed in the first day of life by NIRS and head ultrasound. All patients were transferred in stable respiratory and hemodynamic conditions to the cath lab, OR, or ICU. The patient who required ECMO support in the DR had a positive clinical and surgical outcome. Survival to discharge for our entire cohort was 85% (six deaths/40). As for the most represented groups of patients, CDH and dTGA, survival to discharge was, respectively, 86% (two deaths/14) and 100%. The mortality cases were related to severe pulmonary hypoplasia in two high-risk CDH patients after surgical hernia repair and VV-ECMO support, intractable hypotension in one patient with renal dysplasia who underwent bilateral nephrectomy, and multiorgan failure after atrial septostomy and DA stent placement in a premature infant with PAIVS and hydrothorax; one death occurred in the OR during surgical correction of TAPVR and was related to severe pulmonary vein hypoplasia. Palliative care was offered to a patient with cerebral tumor.

## Discussion

This report provides our most recent experience as a tertiary care referral center for high-risk pregnancies where an advanced DRICU model has been implemented. Most of the cases are newborns with high-risk CHD, CDH, and hydrothorax/fetal hydrops. High-risk CHD requiring immediate or emergency intervention and in whom severe instability is anticipated have been clearly defined by Pruetz et al. (4). The most frequent diagnoses are dTGA with restrictive FO, obstructed TAPVR, or HLHS with restrictive atrial septum. Isolated dTGA is the most common cyanotic CHD; inadequate intracardiac mixing and profound hypoxemia may occur at birth with rapid and severe deterioration. Fetal predictors of inadequate circulatory mixing have been described: atrial septal features, such as restrictive, hypermobile, and redundant FO, were significantly correlated with the need for urgent BAS at birth (5). In obstructed TAPVR and other types of CHD, cardiorespiratory compromise may be significant immediately after birth, and intensive care assistance is key to allow transition to the cath lab or to the OR in a stable condition. Tachyarrhythmias and complete congenital heart block should also be considered as critical forms of CHD, requiring DRICU for medical therapy and/or emergency pacing (4).

It is common opinion that assistance in the delivery room with intensive care standards has the potential to affect the clinical course and outcome in CDH infants (9). Although guidelines on CDH resuscitation have been published (9–11), consistent treatment pathways in the delivery room are not defined in this population, and clinical practice varies between centers. In our DRICU pathway, we focus our immediate assistance on these objectives: to establish adequate perfusion and oxygenation protecting hypoplastic lungs from high airway pressures and to help transition from fetal to neonatal cardiopulmonary physiology, with early inotropic support and treatment of pulmonary hypertension.

In newborns with hydrothorax or fetal hydrops, respiratory failure can be partially relieved by the planned removal of pleural and/or peritoneal fluid before birth (prenatal thoracentesis/paracentesis), or as soon as the newborn is delivered. Pericardiocentesis is seldom necessary. Avoiding high airway pressures and implementing a “gentle” ventilation strategy may protect from air leak syndromes, lung damage, and hemodynamic instability.

The indications for EXIT include obstructive head/neck/chest masses, congenital high airway obstruction syndrome (CHAOS), and EXIT-to-ECMO for severe CDH patients (6). In perinatal care planning, the high maternal and fetal/neonatal risks related to EXIT should be carefully balanced with benefits. Before 2016, we have planned EXIT in rare cases of CHAOS. In this study, we report two EXIT procedures for tracheal plug removal. Fetal tracheal occlusion promotes lung growth in selected cases of severe CDH by temporarily balloon-occluding the trachea. Fetoscopic procedures are associated with an increased risk of iatrogenic premature rupture of membranes (PROM) in up to 30% of cases (12). The tracheal plug should be removed at 34 weeks GA fetoscopically, but in the case of PROM, the balloon is in place at the time of delivery, and emergency EXIT procedure is mandatory to safely free the airway by tracheoscopy, as in our cases. The procedure EXIT-to-ECMO was developed to provide cardiopulmonary support in severe CDH patients, but its application in this setting remains highly controversial (6). We considered the indication EXIT-to-ECMO in one patient with PAIVS and prenatal closure of DA. With low or absent flow through the pulmonary valve and the DA, we expected early and profound desaturation and cardiocirculatory compromise, not readily treatable with PGE infusion after birth. ECMO support was indicated as a bridge to systemic-pulmonary shunt or DA stenting. In this case, lack of parental consent secondary to the high maternal risks associated with the EXIT procedure led to an alternative strategy. As foreseen, the rapid clinical deterioration, despite PGE infusion, required emergency ECMO cannulation and support, which were rapidly implemented with a percutaneous technique.

We consider this updated concept of DRICU, not yet described in the literature, an effective institutional model that could be applied in tertiary care centers where complex congenital pathologies are managed. The increasing ability in the prenatal detection of high-risk pregnancies and the prenatal discussion in a multidisciplinary team of fetal and perinatal medicine are essential to define successful perinatal care pathways. Experienced neonatal intensivists should have adequate skills to manage difficult airways, severe respiratory diseases, as in CDH, and severe hemodynamic instability, as in critical CHD. Routine procedures (intubation, arterial, central venous access, and chest/peritoneal drain placement) should be completed in a timely manner. Protective ventilation strategies applied in the first minutes of life are strongly recommended in patients with lung hypoplasia. Complex procedures (i.e., EXIT, ECMO) should be planned and implemented when indicated. Skilled neonatal nurses are essential for successful teamwork. Family counseling is of utmost importance to share the implications of the diagnosis and related perinatal management. In our model, the multidisciplinary support to the family allows a continuum of care from pregnancy through delivery and neonatal care to long-term follow-up.

Main challenges include coordination of different services, delivery outside the OB/Gyn department, availability of skilled personnel 24/7 for emergency DRICU, equipment setup, and medical and nursing staff training. Over the years, close collaboration between specialists, growing experience with case briefing and debriefing, and continuous education led to a steady improvement of the DRICU model.

Our experience highlights that, even in the most challenging cases, morbidity and mortality are potentially prevented with the DRICU procedure. We report no severe acute complications nor deaths in the delivery room nor in the immediate postnatal period despite complex and very high-risk conditions.

It is difficult to comment on mortality rates related to different pathologies in this limited case series. Deaths occurred in the subsequent clinical course and were not directly related to perinatal assistance. In a recent retrospective analysis, the mortality rate in isolated dTGA after BAS was 6.3% (13), while we report a 100% survival to discharge. Overall survival in this series of CDH patients was 85% despite the fact that half of the patients had poor prognostic factors (right CDH or left CDH with indication to FETO) or required ECMO after birth. The overall survival rates in CDH in recent reports are 50–70%, up to 90% in less severe defects (9, 11, 12).

The study limitations include the retrospective design, the sample size, and the heterogeneity of the diagnoses. The lack of a control group is a limitation. This model was gradually developed before 2016 with increasing improvements in the organizational details. This prevents the identification of a defined control group, before and after the implementation of the DRICU model. The aim of this model is to provide the best standard of care in a selected cohort of conditions associated with a known high risk of complications in terms of morbidity (air leak syndromes, refractory hypoxemia, hemodynamic instability, and acute neurological complications) and mortality in the delivery room. Despite these limitations, our opinion is that an advanced DRICU model may provide the highest level of care and may improve clinical outcomes in newborns with complex pathologies and high risk of morbidity and mortality. Further evidence is definitely needed to define optimal perinatal pathways, management strategies, and resuscitation procedures in this setting.

### Future Developments

In the past decade, we brought intensive care in the DR. The next frontier in the DRICU approach will be to have the newly born at the center of a highly technological OR where delivery is planned. The OR should be integrated with multi-plan angiography and intraoperative MR. In this facility, which is in advanced development in our institution, the critical newborn could benefit, immediately after birth, of complex surgeries, cardiac catheterization with eventual hybrid approach, MR study, and neuroradiological procedures.

## Conclusions

In high-risk newborns, planned assistance strategies and specialized competencies may improve the outcome and prevent severe complications shortly after birth. As recommended by Vento, “intensive care of newborns at the highest risk begins antenatally and should be continued with equal or greater attention throughout transition in the DR and into the NICU” (1). Highly specialized approaches have been developed in the past few years, but we expect further advances to improve resuscitation and stabilization procedures in complex newborns.

## Data Availability Statement

The original contributions presented in the study are included in the article/supplementary material, further inquiries can be directed to the corresponding author/s.

## Ethics Statement

The study involving human participants was reviewed and approved by Istituto Giannina Gaslini Institutional Review Board (n. 0011644/20). Written informed consent from the participants' legal guardian/next of kin was not required to participate in this study in accordance with the national legislation and the institutional requirements.

## Author Contributions

The design of the study was formulated by the lead author, SB with AM. An initial literature review was performed by SB and IB and further updated by all co-authors. The data collection was completed by IB, MF, and EL. This work was critically revised for important intellectual content and received final approval of the version to be published by all co-authors. All the authors agreed to be accountable for all aspects of the work, ensuring that questions related to the accuracy or integrity of any part of the work are appropriately investigated and resolved. All the co-authors were involved in the design and drafting of the paper.

## Conflict of Interest

The authors declare that the research was conducted in the absence of any commercial or financial relationships that could be construed as a potential conflict of interest.
